# Mechano-growth factor accelerates the proliferation and osteogenic differentiation of rabbit mesenchymal stem cells through the PI3K/AKT pathway

**DOI:** 10.1186/s12858-015-0031-z

**Published:** 2015-01-15

**Authors:** Yanxiang Tong, Wei Feng, Yimin Wu, Huicheng Lv, Yanfei Jia, Dianming Jiang

**Affiliations:** Department of Orthopedic, The First Affiliated Hospital of Chongqing Medical University, Chongqing, China; The Second Affiliated Hospital of Inner Mongolia Medical University, Hohhot, China

**Keywords:** rMSCs, PI3K/AKT, MGF, Osteogenic differentiation

## Abstract

**Background:**

Mesenchymal stem cells (MSCs) can differentiate into chondroblasts, adipocytes, or osteoblasts under appropriate stimulation. Mechano-growth factor (MGF) reportedly displays a neuroprotective effect in cerebral regions that were exposed to ischemia and is expressed in stromal cells of the eutopic endometrium and in glandular cells of the ectopic endometrium.

**Results:**

This study sought to understand the potential involvement of phosphatidylinositol-3-kinase (PI3K)/protein kinase B (AKT) in MGF-induced growth of rabbit MSCs (rMSCs). We applied various concentrations of MGF to cultured rMSCs and observed the growth rate of the cells, the changes in the phosphorylation state of AKT and mammalian target of rapamycin (mTOR), and the expression levels of alkaline phosphatase and osteocalcin. We found that the growth and osteogenic differentiation of MGF-induced rMSCs were promoted primarily by phosphorylated AKT, and that this phosphorylation, as well mTOR phosphorylation, was mediated by the MGF receptor.

**Conclusion:**

Our study suggests that MGF promotes the growth and osteogenic differentiation of rMSCs primarily through the PI3K/AKT pathway.

## Background

Mesenchymal stem cells (MSCs) can differentiate into chondroblasts, adipocytes, or osteoblasts under appropriate stimulation [1]. Adult rabbit MSCs (rMSCs) are an important source for tissue repair and therapy in regenerative medicine [2]. Therefore, expanding the osteogenic capacity of rMSCs is of major interest for improving the osteogenic potential of rMSCs for optimal bone regeneration [3]. The osteogenic differentiation of MSCs is characterized by the appearance of timely expressed genes, such as runt-related transcription factor 2, alkaline phosphatase (ALP) and type I collagen, followed by extracellular matrix mineralization [4].

Phosphatidylinositol-3-kinase (PI3K)/protein kinase B (AKT) signaling pathways are activated by a variety of extracellular stimuli and regulate a wide range of cellular processes, including cell motility, survival, proliferation, and cell cycle progression. Recent studies showed that the activation of PI3K/AKT is involved in cell survival and axonal outgrowth in neurons [5-8]. Mechano-growth factor (MGF) has been found in many tissues. It reportedly displays a neuroprotective effect in cerebral regions that were exposed to ischemia and is expressed in stromal cells of the eutopic endometrium and in glandular cells of the ectopic endometrium. In recent years, increasing attention has been paid to MGF because of its regenerative effects on neurons, human mesenchymal stem cells, and osteoblasts [9,10]. In addition, tissue-protective actions of MGF have been demonstrated in acute myocardial infarction, neuron damage, and rabbit bone-defect models [11].

Here, we used MGF to investigate the molecular mechanisms underlying MSC osteoblast differentiation and enhance the osteogenic potential of rabbit MSCs. Our data revealed that MGF through PI3K/AKT signaling pathways trigger osteogenic differentiation of rabbit bone marrow-derived mesenchymal stromal cells.

## Results

### Identification of rMSCs

**Figure 1:**
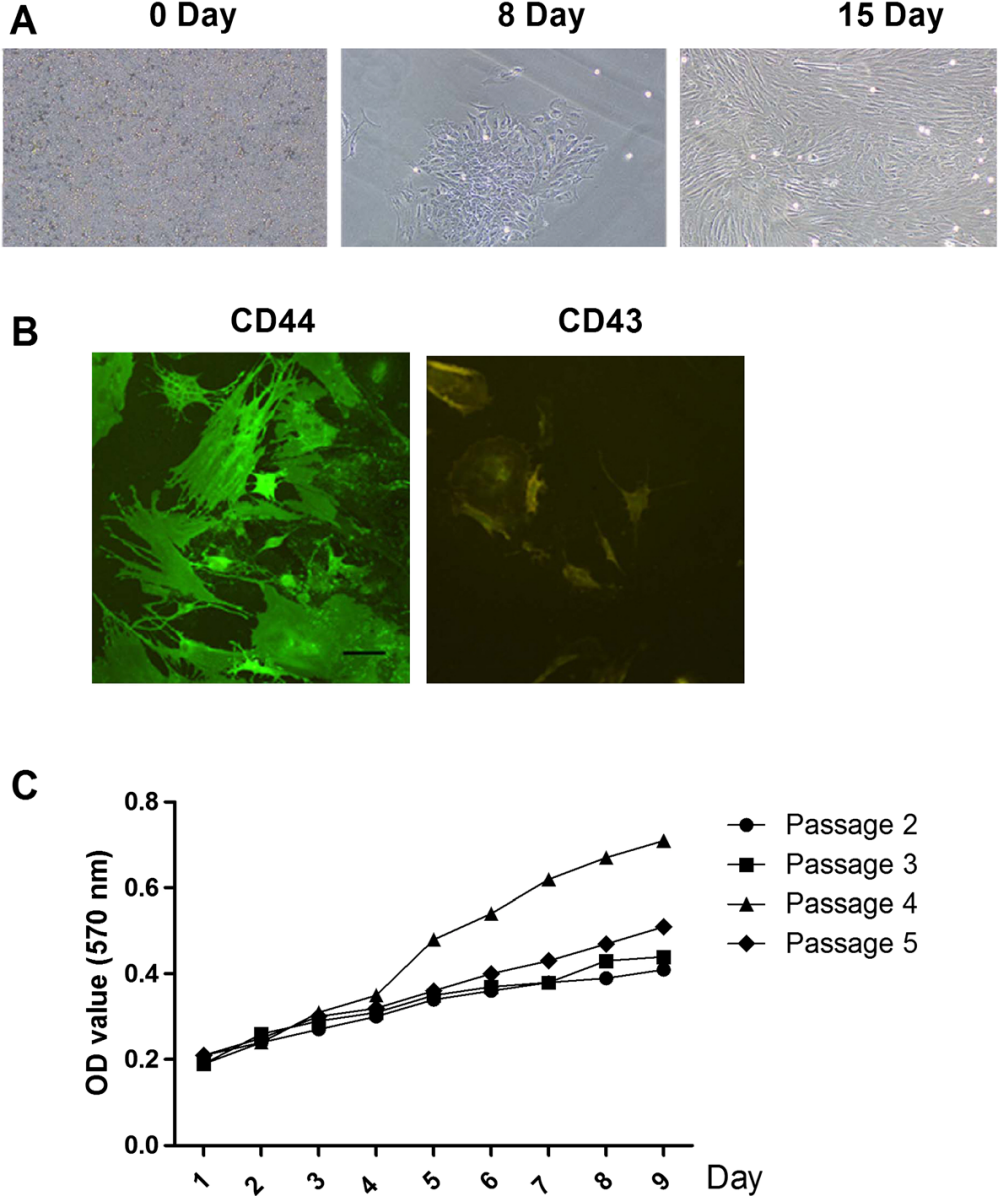
**Identification of rMSCs. (A)** Morphology of rMSCs at 0, 8, and 15 days of culture. **(B)** Immunofluorescence staining showing the expression of CD34 and CD44 in cultured rBMSCs. **(C)** Cellular proliferation during the second through fifth cell passages (as determined at an absorbance of 570 nm).

The morphology of the rMSCs was determined using a microscope (Figure 1A). At day 10, cells reached 80% confluence. At day 13, the cells displayed a uniform spindle shape and reached 100% confluence. To further identify the rMSCs, CD34 and CD44 cell markers were examined. Immunofluorescence staining indicated that the CD44 cell marker fluoresced green on the cell surface under a fluorescent microscope, whereas CD34 fluoresced brown (not green), indicating that cultured cells were positive for CD44, but negative for CD34 (Figure 1B).

The proliferation of the cells for the second through the fifth cell passages was analyzed with an automated microplate reader at an absorbance of 570 nm on days 1–10. As shown in Figure 1C, the growth curves for the passaged cells displayed “S” shapes, with the latent growth phase of the passaged cells stabilizing during the first to second days. The cells from the fourth and fifth passages were 100% confluent at 7 days and then entered a lag phase. However, the cells from the second and third passages entered the lag phase 2 days later. Thus, the cells from the fourth passage displayed the greatest ability to proliferate (P < 0.05).

### MGF receptor expression in rMSCs

**Figure 2:**
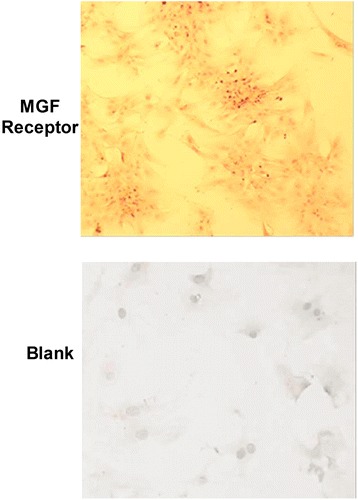
**Mechano-growth factor (MGF) receptors expressed in cultured rBMSCs.**

To detect the expression of MGF receptor in rMSCs, we used immunocytochemistry techniques, and Figure 2 shows MGF receptors expressed in rMSCs. MGF expressed in the cytoplasm.

### Optimal MGF concentration and application time for MGF-induced effects on rMSCs

**Figure 3:**
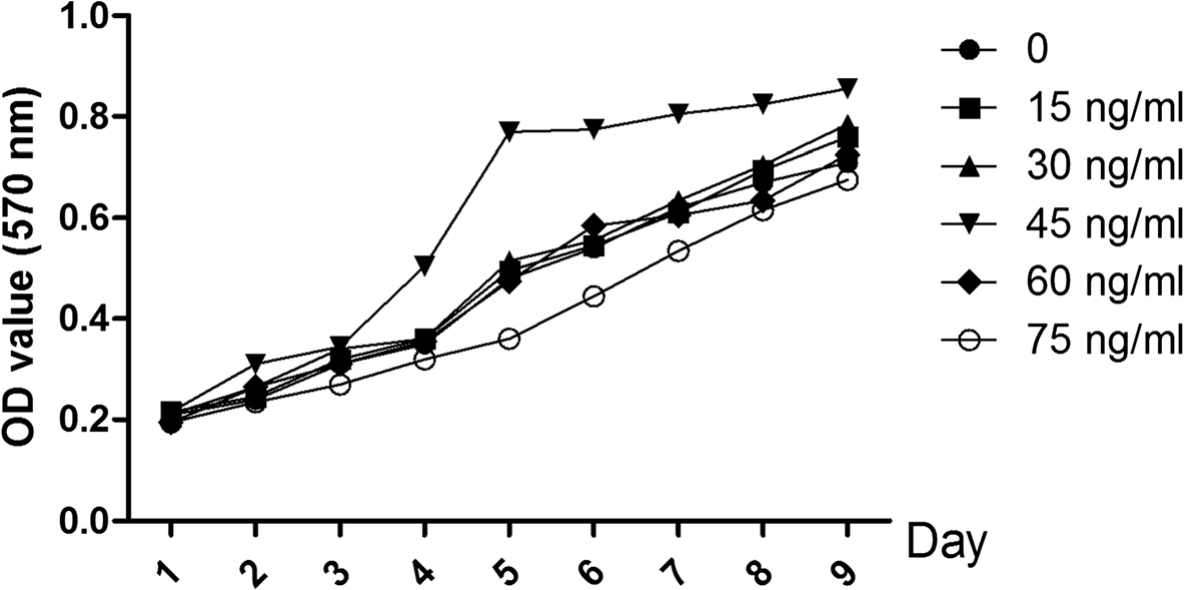
**Growth rates of cultured rMSCs treated for 1–10 days with MGF (15, 30, 45, 60, or 75 ng/ml).**

The optimal MGF concentration and application time for the MGF-induced effects on the rMSCs were determined. Figure 3 shows the growth rates of rMSCs from the fourth cell passage for 1–10 days following the addition to the cell culture media of 15, 30, 45, 60, or 75 ng/ml of MGF. The maximum growth rate appeared at an MGF concentration of 45 nM between days 4 and 5.

### MGF promotes the growth and differentiation of rMSCs via the PI3K/AKT pathway

**Figure 4:**
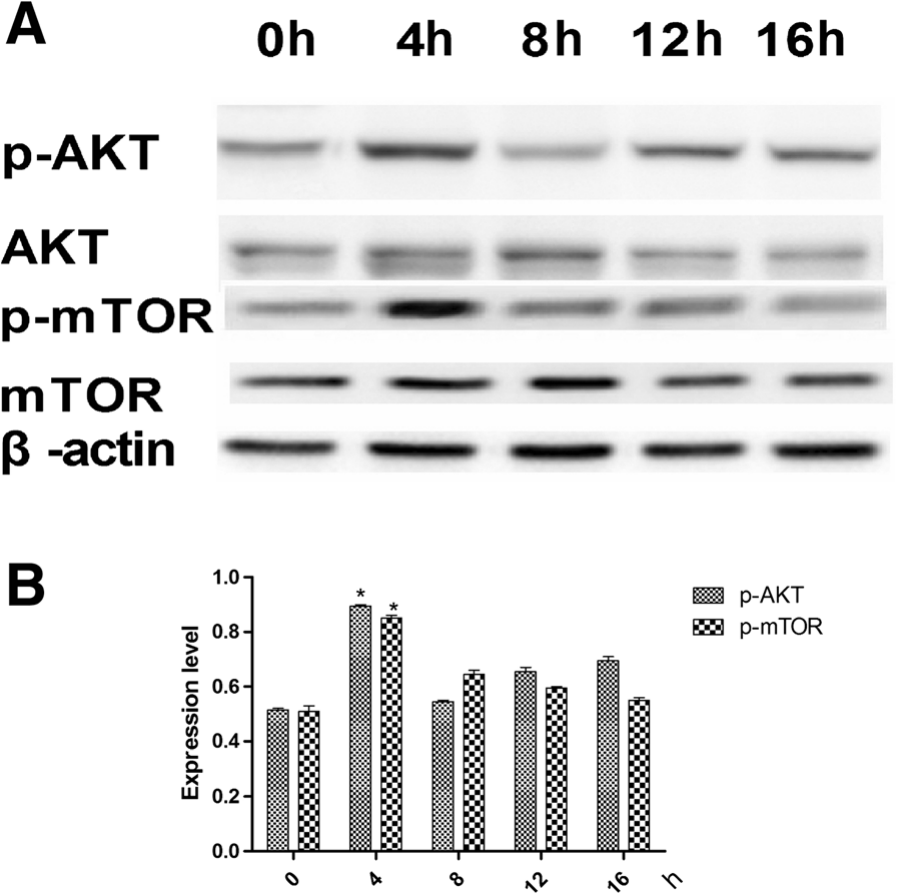
**Effects of MGF (45 nM) on the phosphorylation of AKT and mTOR at 0, 4, 8, 12, and 16 h on the 5th day of MGF treatment. (A)** The expression levels of AKT, mTOR, phospho-AKT, and phospho-mTOR. **(B)** Contrast gray values for the phosphorylation of AKT and mTOR (*P < 0.05).

**Figure 5:**
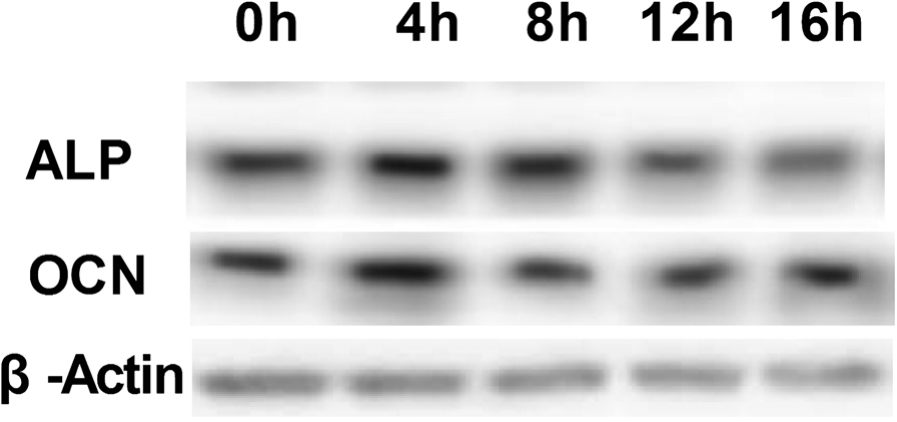
**Expression of ALP and OCN protein levels in 0, 4, 8, 12 and 16 h.**

To identify the signaling pathway through which MGF acts on rMSCs, cells were treated with 45 nM of MGF for 5 days (the optimal conditions as determined above), and the phosphorylation states of AKT and mTOR were examined at 0, 4, 8, 12, and 16 h following treatment on day 5 (Figure 4A). Figure 4B shows that the phosphorylation of AKT and mTOR increased. To further explore the function of MGF, the expression levels of ALP and OCN were examined in the osteoblasts that were formed from the rMSCs. The expression of these two proteins was higher at 4 h that at other time in cells treated with 45 nM MGF for 4 h on the fifth day of treatment (Figure 5).

**Figure 6:**
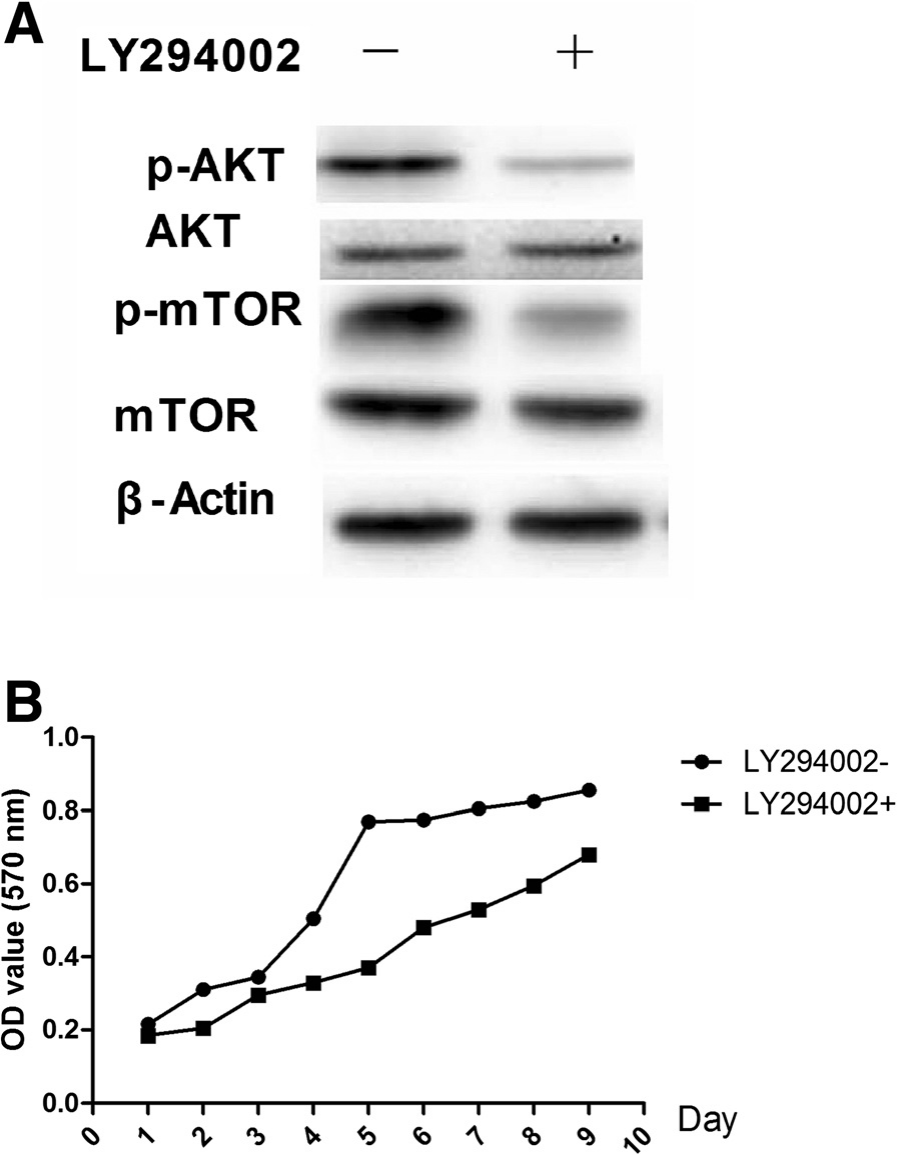
**Effects of LY294002 on the PI3K/AKT pathway and growth rates of rBMSCs. (A)** The protein expression levels of AKT and mTOR and phosphorylated AKT and phosphorylated mTOR following the addition of the PI3K/AKT inhibitor LY294002. **(B)** Effects of LY294002 on the growth rates of rMSCs treated with MGF (45 ng/ml) for 1–10 days.

**Figure 7:**
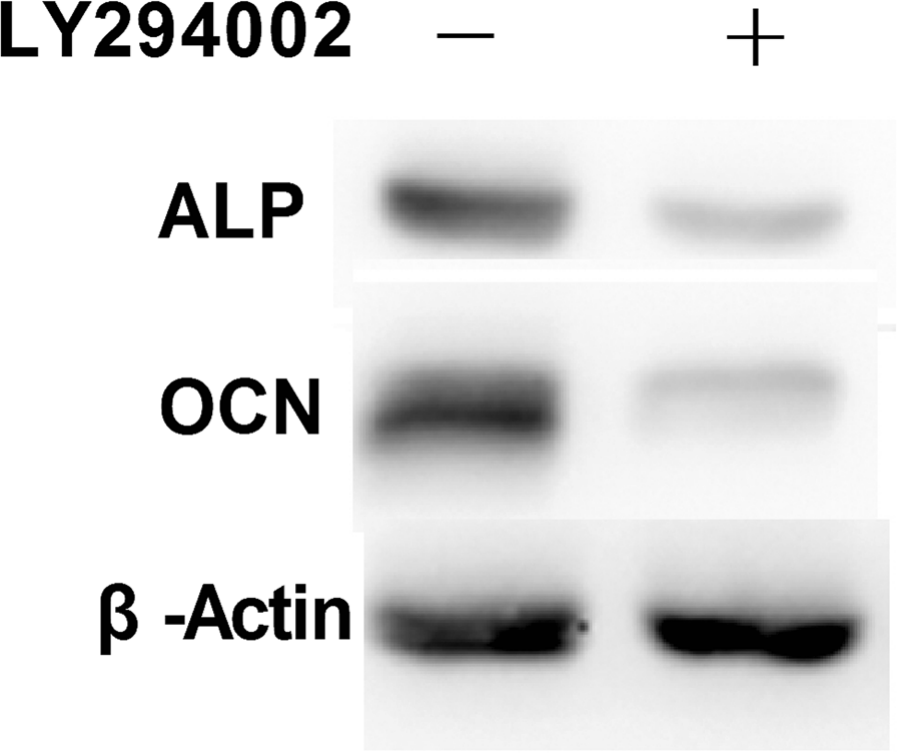
**Effects of LY294002 on the expression of ALP and OCN protein levels.**

To verify that MGF promotes the growth and differentiation of rMSCs via the PI3K/AKT pathway, the PI3K/AKT inhibitor LY294002 with 20 μmol/L was used to inhibit PI3K/AKT activity (Figure 6A). Following the addition of LY294002, the cells were treated with MGF for 1–10 days, and their growth rates were compared with those of cells that did not receive LY294002 treatment (Figure 6B). We found that the phosphorylation of mTOR was decreased compared with that of controls (Figure 6A). The inhibition of PI3K/AKT also blocked the ability of MGF at 45 nM to increase the growth rate of rMSCs, even after 10 days of treatment. In addition, the expression levels of ALP and OCN were decreased compared with those in control osteoblasts (Figure 7).

## Discussion

Finding mechanisms that trigger the growth and osteogenic differentiation of rMSCs may help develop novel therapeutic approaches to promote bone formation [12]. In the present study, we demonstrated that MGF promoted the growth rate and osteogenic differentiation of rMSCs via the PI3K/AKT pathway.

The identity of rMSCs were verified by their morphology and the finding that the rMSCs expressed CD44, a marker of rMSCs, but not CD34, a closely related molecule that does not label rMSCs. Cellular proliferation following the second through fifth passages was analyzed on days 1–10 following the passages. The fourth generation rMSCs showed the greatest capacity for proliferation, whereas the second generation showed the weakest.

We determined that the maximum growth rate of rMSCs was achieved by the addition of 45 nM MGF to the culture medium for 5 days. We also demonstrated that the MGF receptor is expressed at high levels in rMSCs.

To gain further insight into the mechanisms through which MGF promotes growth and differentiation in rMSCs, we evaluated the activity of the signaling pathways downstream of the MGF receptor. The PI3K/AKT/mTOR signaling pathway plays a crucial role in multiple cellular processes, including cell proliferation, angiogenesis, metabolism, differentiation, and survival [13]. The results of our present study suggested that MGF promoted the growth of rMSCs via the PI3K/AKT signaling pathway. We found that 4 h on the fifth day of MGF treatment was required to stimulate maximal phosphorylation of AKT and mTOR and maximal expression of ALP and OCN. Furthermore, inhibiting AKT with LY294002 suppressed the MGF-mediated promotion of growth and osteogenic differentiation in rMSCs.

## Conclusion

Overall, our study suggests that the MGF receptor signaling pathway accelerates growth in rMSCs primarily through a PI3K/AKT-dependent pathway. Despite this promising finding, further study is necessary before any clinical applications can be considered.

## Methods

Unless otherwise specified, all chemicals and reagents were purchased from the Sigma Chemical Company (St. Louis, MO, USA). MGF was purchased from Chemicon International (Temecula, CA, USA). Antibodies to IgG, β-actin, AKT, mammalian target of rapamycin (mTOR), ALP, osteocalcin (OCN), CD44, CD34, MGF receptor, phospho-AKT, phospho-mTOR, and phospho-p90RSK1 (Ser380) were purchased from the Millipore Corporation, USA.

### Isolation and culture of rMSCs

The femur from a neonatal New Zealand white rabbit was isolated, and the ends of the femur were opened. The bone marrow was flushed from the femur with low glucose Dulbecco’s modified Eagle’s medium (DMEM) using a 1 mL syringe. Cells were harvested in a culture dish, dissolved using a Pasteur pipette, seeded onto a flask containing DMEM and 15% fetal bovine serum, and cultured in an incubator with 5% CO2 at 37°C. The medium was replaced every 2 days. When cells grew to a confluence of approximately 85%, they were passaged with 0.25% trypsin and 0.1% EDTA (1:2). Cell growth was monitored under an inverted phase contrast microscope (Nikon Co.). The animal test was agreed by The Chongqing Medical University Experimental Animal Management Committee.

### 3-(4,5-Dimethylthiazol-2-yl)-2,5-diphenyltertrazolium bromide (MTT) assay

Cells were grown in 96-well plates (1 × 10^3^ cells/well) supplement with MGF. Control cells were switched from RPMI-1640 to DMEM containing 0.1% dimethyl sulfoxide (DMSO). At 1, 2,3, 45,6,7,8 and 9 days following MGF treatment (0, 15, 30, 45, 60 and 75 nM MGF), 20 μL of MTT was added to each well to a final concentration of 0.5%. After a 4 h incubation at 37°C in the dark, 150 μL DMSO was added to each well for 10 min to dissolve the formazan crystals. The absorbance was measured using a microplate reader (EXL800, Cole-Parmer, Vernon Hills, IL, USA) at 490 nm. All experiments were repeated three times. The viability of the MGF treated cells was expressed as percentage of population growth plus the standard error of the mean (SEM) relative to that of untransfected control cells. Cell growth was calculated as follows: % growth = (mean experimental absorbance- mean control absorbance/mean control absorbance) × 100.

### Immunofluorescence

The rMSCs were fixed in 3.7% paraformaldehyde for 30 min at room temperature, permeabilized with 0.5% Triton X-100 in PBS for 15 min, and blocked with 1% BSA in phosphate buffered saline (PBS) with 10% goat serum overnight at 4°C. The samples were then stained with primary antibodies diluted in PBS. The primary antibody binding was detected with an Alexa Fluor 488 Goat anti-rabbit IgG (H + L) secondary antibody. Images were captured with a Nikon A1 confocal microscope. Experiments were performed in triplicate.

### Immunohistochemistry

Immunohistochemistry was performed as follows: cell sections were three times for 1 min in phosphate buffered saline (PBS), once fixed for 15 min in ice acetone, three times for 2 min in PBS, 0.5% Triton X-100 20 min. Antigens were recovered by heating the sections in a microwave oven for 15 min, after which the sections were washed three times for 5 min with PBS. Endogenous peroxidase activity was blocked by soaking the slides in a solution of 3% hydrogen peroxide for 15 min at room temperature (RT), followed by washing three times for 5 min with PBS. Non-immune Goat blood serum (50 μL) was added to each section for 15 min at RT, followed by the primary antibodies (Millipore, 50 μL). Slides were incubated overnight at 4°C in a humidified chamber and washed three times for 10 min in PBS. Biotin-labeled secondary antibodies (50 μL) were added to each section and kept for 15 min at 37°C, followed by washing three times for 5 min with PBS. Samples were incubated for 15 min at RT and washed three times for 5 min in PBS before the addition of 100 L freshly prepared 3,3-diaminobenzidine (DAB) for approximately 5–20 min. The reaction was stopped by washing in cold water. Slides were counterstained with hematoxylin, followed by a sealing procedure using neural gum.

### Western blot

The protein homogenates from human neurons were separated using electrophoresis on 8-12% sodium dodecyl sulphate/polyacrylamide gels and transferred to immunoblot nitrocellulose membranes. Membranes were blocked for 30 min at room temperature in PBS buffer containing 5% fat-free milk and 0.1% Tween 20. Membranes were then incubated with primary antibody for at least 1 h at room temperature or overnight at 4°C. The membranes were subsequently washed three times with PBS containing 0.1% Tween 20, incubated with peroxidase-conjugated secondary antibodies, and developed using ECL reagents (Pierce, Rockford, IL, USA).

### Osteogenic differentiation

The rMSCs were plated at a density of 5000 cells/cm^2^ and exposed to standard differentiation-inducing media for 21 days. The medium was changed twice per week. Osteogenic differentiation was achieved following standard in vitro protocols. Endothelial differentiation was stimulated by culturing the cells in Endothelial Growth Medium-2 (EGM-2).

### Statistical analysis

Statistically significant differences between gene expression levels were determined using one-way analysis of variance (ANOVA) followed by a Newman–Keuls test with GraphPad Prism version 5 software (GraphPad Software, La Jolla, CA, USA, www.graphpad.com/company/). Replicates were included in the statistical model. Differences were considered statistically significant at the 95% confidence level (P < 0.05). Data are presented as mean ± S.D.

## Abbreviations

MSCs: Mesenchymal stem cells
MGF: Mechano-growth factor
PI3K/AKT: Phosphatidylinositol-3-kinase /protein kinase B
rMSCs: rabbit MSCs
